# *Helicobacter pylori* Infection Is a Risk Factor for Severe Hyperemesis Gravidarum Requiring Prolonged Hospitalization

**DOI:** 10.1111/jog.70099

**Published:** 2025-10-06

**Authors:** Ryuhei Kurashina, Masafumi Toyoshima, Jun Ogawa, Youhei Tsunoda, Nozomi Ouchi, Mirei Yonezawa, Rintaro Sawa, Yoshimitsu Kuwabara, Shunji Suzuki

**Affiliations:** ^1^ Department of Obstetrics and Gynecology Nippon Medical School, Musashikosugi Hospital Kawasaki city Kanagawa Japan; ^2^ Department of Obstetrics and Gynecology Nippon Medical School Bunkyo‐ku Tokyo Japan

**Keywords:** *Helicobacter pylori*, hyperemesis gravidarum, preconception care, prolonged hospitalization, urinary ketones

## Abstract

**Objective:**

This retrospective study investigated the relationship between *Helicobacter pylori* ( *H. pylori* ) infection and prolonged hospitalization in patients with severe hyperemesis gravidarum (HG). We also aimed to identify other factors associated with extended hospital stays.

**Methods:**

We analyzed data from 164 patients with severe HG. The patients were initially divided into two groups based on their anti‐ *H. pylori* IgG antibody status (positive vs. negative). Subsequently, a second analysis stratified all patients into a short‐stay group (< 21 days) and a long‐stay group (≥ 21 days).

**Results:**

Patients who were positive for anti‐ *H. pylori* IgG antibodies had significantly longer hospital stays (median 24 vs. 15 days, *p* = 0.032). In a univariate analysis, anti‐ *H. pylori* IgG positivity, higher serum free thyroxine (FT4) levels, and strong urine ketone positivity were all significantly associated with long‐term hospitalization. In the subsequent multivariate analysis, anti‐ *H. pylori* IgG positivity emerged as an independent risk factor for prolonged hospitalization (odds ratio 4.67, 95% CI 1.61–13.49, *p* = 0.004). Although strongly positive urine ketones were also associated with longer hospital stays (median 19 vs. 12 days, *p* = 0.001), this was not identified as an independent risk factor (odds ratio 2.169, 95% CI 0.97–4.85, *p* = 0.059).

**Conclusion:**

Our findings suggest that testing for anti‐ *H. pylori* IgG antibodies may help identify patients at a higher risk for a longer hospital stay due to severe HG. Pregnant women who test positive for anti‐ *H. pylori* IgG antibodies may benefit from closer monitoring. Additionally, these results raise the possibility that preconception care could be a preventive measure.

## 1 Introduction

Morning sickness, characterized by nausea, vomiting, and altered food preferences, is a common condition during the first trimester, affecting 45%–90% of pregnant women [1]. Many patients also report increased saliva secretion, changes in taste, and general malaise [2]. Additionally, symptoms such as dry skin due to dehydration, palpitations, oliguria, and insomnia can occur [3]. These symptoms are typically transient, peaking between 7 and 12 weeks of gestation, and most resolve spontaneously by 12–16 weeks [2].

Hyperemesis gravidarum (HG), a severe form of morning sickness, complicates approximately 0.3%–2% of pregnancies [4]. The diagnosis of HG is typically made based on criteria including almost daily vomiting, strongly positive urine ketones, and persistent weight loss, especially a weight loss of 5% or more of pre‐pregnancy body weight [5]. HG is the most common cause of hospitalization in the first half of pregnancy and the second most common cause of hospitalization (after preterm labor) throughout pregnancy [6, 7]. Of patients hospitalized for HG, approximately 10% experience prolonged symptoms throughout their pregnancy [8]. As the disease progresses, electrolyte and acid–base imbalances may occur, leading to liver and kidney dysfunction and impaired consciousness [9]. Furthermore, Wernicke's encephalopathy caused by vitamin B1 deficiency can lead to neurological sequelae [10]. If Wernicke's encephalopathy is not effectively treated, there is an increased risk of its transition to Korsakoff's syndrome, characterized by impaired memory, disorientation, and deficits in speech production [11]. Therefore, prompt initiation of treatment is crucial to prevent the development of these complications [12].

Several theories have been proposed for the etiology of morning sickness: one plausible theory implicates human chorionic gonadotropin (hCG), a hormone secreted by the chorionic villi that peaks around the 10th week of gestation [13]. HG is more common in primiparous women than in multiparous women. Other risk factors include young maternal age, conditions associated with high placental volume (e.g., hydatidiform mole and multiple gestations), genetic predisposition, history of malaria, and female fetal sex [14]. In addition to these factors, *Helicobacter pylori* ( *H. pylori* ) infection has recently been reported as a significant risk factor for developing HG [15, 16, 17, 18].

Worldwide, *H. pylori* infection rates vary widely due to differences in sanitation, diet, and lifestyle in different regions. In Japan, the infection rate of *H. pylori* is higher in the elderly population. This contrasts with decreasing infection rates in younger generations, a trend attributed to improvements in sanitation and lifestyle. A cross‐sectional study that included 14 716 Japanese individuals aged 20 years or older reported the overall prevalence of *H. pylori* infection was 37.6% in women [19]. However, limited data are available regarding the association between *H. pylori* infection and the duration of severe illness or hospitalization for HG in the Japanese population. In this study, we analyzed patients with severe HG who required hospitalization and investigated whether prolonged hospitalization can be predicted using several parameters at the time of admission in the Japanese populations.

## 2 Materials and Methods

### 2.1 Ethics Statement

This study was approved by the Ethics Committees of Nippon Medical School Hospital (approval number: B‐2022‐573) and Nippon Medical School Musashikosugi Hospital (approval number: 715‐5‐26).

### 2.2 Consent

We utilized an opt‐out consent process, a method of obtaining consent that is widely used in Japan for observational studies using existing data. As there were no patients who opted out, all patient data were included in the analysis. No written consent has been obtained from the patients, as there is no patient identifiable data included in the manuscript.

This was a multicenter, retrospective, case–control study. The study enrolled 164 patients with severe HG who required hospitalization at Nippon Medical School Hospital or Nippon Medical School Musashikosugi Hospital from January 2011 to June 2023. A retrospective analysis was performed using their medical records. Hospitalization criteria included the inability to tolerate oral fluids, a 5% or greater reduction in pre‐pregnancy body weight, and strongly positive urine ketones.

Upon admission, all patients received standardized supportive care in accordance with institutional protocols. This consisted primarily of intravenous fluid resuscitation to correct dehydration and electrolyte imbalances. To prevent nutritional deficiencies, thiamine and other vitamin supplements were routinely administered. Antiemetic therapy was not administered routinely; however, if pharmacological treatment was deemed necessary for persistent nausea and vomiting, metoclopramide was used as the first‐line agent. Patient progress was monitored daily through assessment of body weight, oral intake volume, and urine ketone levels. Patients were discharged when they could tolerate at least 50% of their meals without vomiting.

Assessed patient characteristics and clinical data included: age, parity, gestational age at admission, history of psychiatric illness, history of infertility treatment, weight loss from pre‐pregnancy baseline, qualitative urine ketone testing, anti‐ *H. pylori* IgG antibody status, complete blood count, biochemical parameters, thyroid function tests, and duration of hospitalization. All laboratory data were collected upon admission. The raw data used for the statistical analysis are provided as Dataset S1.

**FIGURE 1:**
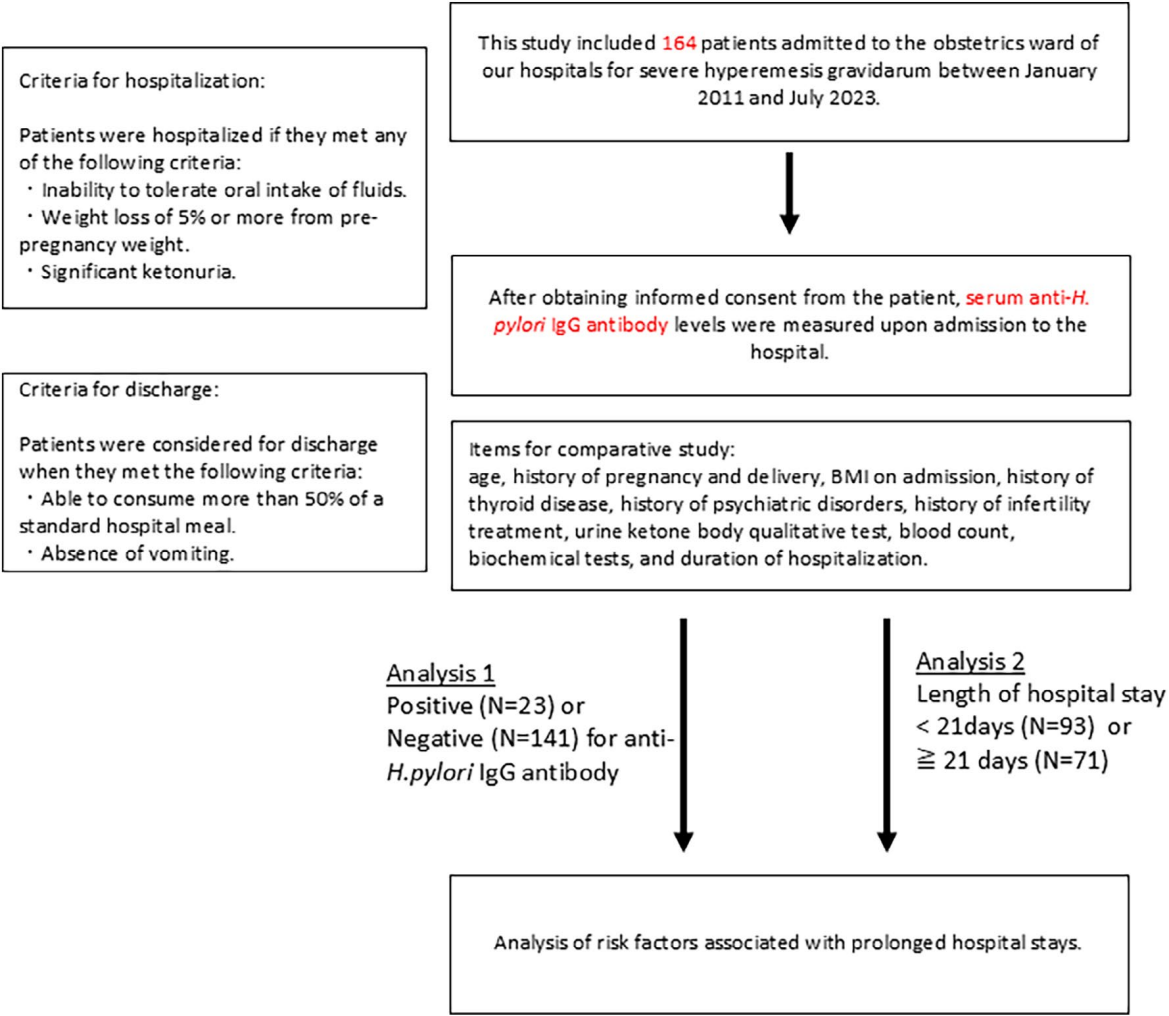
Study flowchart illustrates the patient selection process, criteria for hospitalization, criteria for discharge, and data collection/analysis items for this study. Serum anti‐ *H. pylori* IgG antibody levels were measured upon admission to the hospital. Analysis of risk factors associated with prolonged hospital stays was conducted based on the presence (*N* = 23) or absence (*N* = 141) of anti‐ *H. pylori* IgG antibody, and the length of hospital stay (< 21 days (*N* = 93) or ≥ 21 days (*N* = 71)).

First, patients were divided into two groups based on the presence or absence of anti‐ *H. pylori* antibodies, and these groups were compared to assess for differences in each parameter. Subsequently, prolonged hospitalization was defined as a hospital stay of 21 days or more. This cutoff was selected based on clinical experience that a typical hospitalization for severe HG lasts for approximately 2–3 weeks, and stays exceeding this period are considered prolonged and require more intensive management. The patients were then further categorized into two groups: those with a hospital stay of less than 21 days and those with a hospital stay of 21 days or more. These two groups were then similarly compared to assess for differences in each parameter. The study flow chart is shown in Figure 1. Variables for the multivariate logistic regression analysis were selected based on their significant association with prolonged hospitalization in the univariate analysis, in addition to other established clinical factors.

Statistical analyses were conducted using IBM SPSS Statistics version 29.0.2 (IBM Corp., Armonk, NY, USA). Mann–Whitney *U* tests, *t*‐tests, Fisher's exact tests, and logistic regression analyses were employed as appropriate. *p* values less than 0.05 were considered statistically significant.

## 3 Results

Of the 164 patients, 23 (14.0%) were positive for anti‐ *H. pylori* IgG antibodies, and 141 (86.0%) were negative. Patient profiles and laboratory findings on admission were compared based on anti‐ *H. pylori* IgG antibody status (positive vs. negative) (Table 1). The comparison revealed that hyperthyroidism was significantly more common in the anti‐ *H. pylori* IgG‐positive group. Regarding blood test results, D‐dimer levels were significantly higher in the anti‐ *H. pylori* IgG‐negative group. No significant differences were found in other parameters between the two groups.

**TABLE 1:** Patient characteristics and laboratory data stratified by anti‐ *H. pylori* IgG antibody presence.

	Anti‐*H. pylori* IgG antibody		*p*
	Negative (*N* = 141)	Positive (*N* = 23)	
Primigravida	69.2%	70.0%	0.804
Nulliparous	42.6%	47.8%	0.779
ART	26.9%	33.3%	0.727
History of mental illness	7.1%	4.5%	1
Hyperthyroidism	4.3%	17.4%	*0.032
Hypothyroidism	7.2%	9.1%	0.67
Urine ketone level of 3+ or higher	69.2%	66.7%	0.804
Age	33 (24–47)	32 (29–45)	0.807
BMI (kg/m^2^)	19.8 (14.3–32.1)	19.0 (16.0–24.2)	0.662
WBC (/μL)	7100 (1700–21 200)	7650 (4100–12 900)	0.054
Hb (g/dL)	12.9 (9.1–16.1)	12.5 (10.5–14.0)	0.138
Hct (%)	37.2 (28.8–46.2)	36.3 (31.5–40.8)	0.187
Plt (/μL)	23.7 (10.2–66.8)	25.0 (13.0–33.5)	0.522
AST (U/L)	22 (10–131)	17 (12–147)	0.557
ALT (U/L)	13 (1–280)	14 (8–485)	0.977
LD (U/L)	145 (63–1137)	149 (103–188)	0.556
γGT (U/L)	13 (4–103)	12 (7–51)	0.405
T‐Bil (mg/dL)	0.7 (0.3–3.6)	0.7 (0.3–3.7)	0.812
BUN (mg/dL)	8.2 (1.7–19.8)	8.6 (3.8–15.9)	0.710
Cre (mg/dL)	0.46 (0.24–0.74)	0.49 (0.32–0.68)	0.242
CRP (mg/dL)	0.05 (0.01–3.78)	0.04 (0.01–0.62)	0.233
D‐dimer (μg/mL)	0.8 (0.4–12.6)	0.65 (0.4–0.7)	*0.006
FT3 (pg/mL)	2.85 (1.45–15.50)	2.61 (1.67–9.22)	0.127
FT4 (pg/mL)	1.520 (0.82–7.78)	1.525 (1.06–4.26)	0.476
TSH (μIU/mL)	0.310 (0.005–4.100)	0.433 (0.005–1.455)	0.427
hCG (mIU/mL)	155686.6 (8063.0–566155.5)	149118.2 (23767.0–277148.0)	0.540

*indicate statistically significant differences (*p* < 0.05).

**FIGURE 2:**
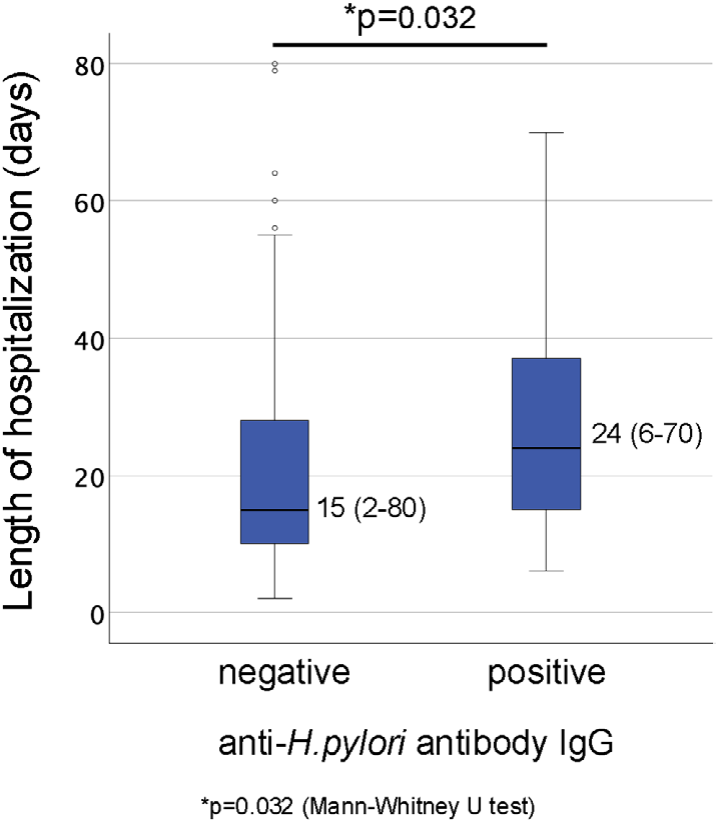
Comparison of median duration of hospitalization (range) between patients with and without anti‐ *H. pylori* IgG antibodies. The Mann–Whitney *U* test was used for statistical analysis, yielding a *p* value of 0.032.

Subsequently, a comparison of the length of hospital stay between these two groups revealed that the median duration of hospitalization (range) was significantly longer for the anti‐ *H. pylori* IgG antibody‐positive group (24 days, range: 6–70) compared to the anti‐ *H. pylori* IgG antibody‐negative group (15 days, range: 2–80) (*p* = 0.032, Figure 2).

As a secondary outcome, we compared patients requiring hospitalization for 21 days or more (long‐term hospitalization group) with those hospitalized for less than 21 days (short‐term hospitalization group). When comparing the two groups, the long‐term hospitalization group had significantly higher proportions of patients with strongly positive urinary ketones (*p* = 0.036) and those positive for anti‐ *H. pylori* IgG (*p* = 0.022) (Table 2). A comparison of laboratory findings on admission revealed that serum free thyroxine (FT4) levels were significantly higher in the group with prolonged hospital stay; however, no significant differences were found in other parameters between the groups (Table 2).

**TABLE 2:** Patient characteristics and laboratory data stratified by prolonged hospitalization.

	Duration of hospitalization		*p*
	Less than 21 days (*N* = 93)	21 days or more (*N* = 71)	
Primigravida	80.6%	84.5%	0.544
Nulliparous	65.5%	67.6%	0.787
ART	28.8%	26.0%	0.736
History of mental illness	7.6%	5.7%	0.758
Hyperthyroidism	5.4%	7.7%	0.746
Hypothyroidism	7.6%	7.2%	0.931
Urine ketone level of 3+ or higher	62.2%	78.1%	*0.036
Anti‐*H. pylori* IgG(+)	8.6%	21.2%	*0.022
Age	33 (24–47)	33 (24–47)	0.407
BMI (kg/m^2^)	19.65 (15.07–32.11)	19.79 (14.30–31.71)	0.806
WBC (/μL)	7100 (2300–21 200)	7300 (2800–11 500)	0.488
Hb (g/dL)	12.8 (10.5–16.1)	12.7 (10.5–14.9)	0.778
Hct (%)	36.6 (30.7–46.2)	36.5 (31.5–43.0)	0.925
Plt (×104/μL)	23.7 (12.9–66.8)	23.7 (10.2–36.2)	0.791
AST (U/L)	18 (11–147)	17 (12–76)	0.807
ALT (U/L)	15 (1–483)	12 (7–229)	0.463
LD (U/L)	145 (106–238)	139 (63–267)	0.823
γGT (U/L)	12 (4–51)	14 (7–103)	0.143
T‐Bil (mg/dL)	0.70 (0.29–1.77)	0.75 (0.39–3.70)	0.072
BUN (mg/dL)	8.0 (2.4–15.9)	7.2 (2.3–16.4)	0.820
Cr (mg/dL)	0.45 (0.27–0.59)	0.45 (0.27–0.59)	0.315
CRP (mg/dL)	0.04 (0.01–3.78)	0.04 (0.01–0.71)	0.654
D‐dimer (μg/mL)	0.8 (0.4–7.8)	0.8 (0.4–12.6)	0.715
FT3 (pg/mL)	2.76 (1.45–8.24)	2.80 (1.69–13.6)	0.469
FT4 (pg/mL)	1.48 (0.82–3.79)	1.62 (1.06–4.26)	*0.027
TSH (μIU/mL)	0.383 (0.005–3.450)	0.277 (0.005–2.940)	0.485
hCG (mIU/mL)	155 404 (8063–566 115)	125 400 (421 49–319 777)	0.856

*indicate statistically significant differences (*p* < 0.05).

**FIGURE 3:**
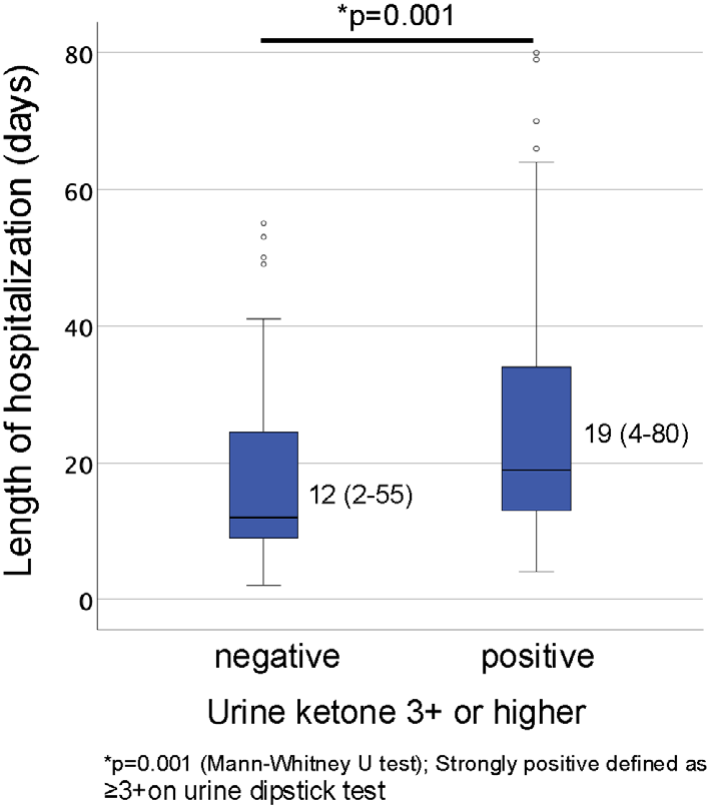
Comparison of median duration of hospitalization (range) between patients with and without strongly positive urine ketone bodies (defined as ≥ 3+ on a urine dipstick test). The Mann–Whitney *U* test was used for statistical analysis, yielding a *p* value of 0.001.

Multivariate logistic regression analysis identified only anti‐ *H. pylori* IgG positivity as an independent risk factor for prolonged hospitalization (odds ratio [OR]: 4.665, 95% Confidence Interval [CI]: 1.613–13.489, *p* = 0.004). Although strongly positive for urinary ketones exhibited a trend toward longer hospital stays, this finding was not statistically significant in the multivariate analysis (OR: 2.169, 95% CI: 0.97–4.85, *p* = 0.059) (Table 3). Conversely, when patients were stratified by the presence or absence of strongly positive urinary ketones, the median length of hospitalization was significantly longer for the positive group (19 days, range: 4–80) compared to the negative group (12 days, range: 2–55) (*p* = 0.001, Figure 3).

**TABLE 3:** Multivariate analysis of factors associated with prolonged hospitalization.

	*β* (SE)	OR	95% CI	*p*
Age	0.052 (0.039)	1.054	0.976–1.138	0.183
Nulliparous	0.221 (0.388)	1.247	0.583–2.669	0.569
Anti‐*H. pylori* IgG(+)	1.54 (0.542)	4.665	1.613–13.489	*0.004
Urine ketone level of 3+ or higher	0.774 (0.411)	2.169	0.97–4.85	0.059
FT4	0.292 (0.244)	1.339	0.831–2.158	0.231
History of mental illness	0.091 (0.698)	1.095	0.279–4.298	0.896

Abbreviations: *β*, regression coefficient; CI, confidence interval; OR, odds ratio; SE, standard error.

*indicate statistically significant differences (*p* < 0.05).

## 4 Discussion

Our study demonstrated that patients positive for anti‐ *H. pylori* IgG antibodies experienced a longer duration of severe HG, requiring more prolonged hospital stays compared to their seronegative counterparts. *H. pylori* infection is a well‐established risk factor for various gastroduodenal diseases, including peptic ulcer disease, chronic atrophic gastritis, and gastric adenocarcinoma [20]. A link between HG and exposure to *H. pylori* has also been reported [18, 21].

One potential mechanism linking *H. pylori* infection to HG is a systemic inflammatory response [22]. The infection triggers chronic gastritis, releasing pro‐inflammatory cytokines like interleukin‐1β, interleukin‐6, and tumor necrosis factor‐α [23]. These can cross the blood–brain barrier, stimulating the chemoreceptor trigger zone and causing nausea and vomiting [24]. Elevated cytokine levels during early pregnancy may also worsen the inflammatory response to *H. pylori* , increasing HG severity [25].

Another plausible mechanism involves *H. pylori* ‐induced gastric dysmotility. *H. pylori* infection has been associated with delayed gastric emptying and impaired gastric accommodation, which may contribute to the nausea and vomiting experienced in HG [26]. The exact mechanisms underlying these effects are unclear but may involve alterations in gastrointestinal hormones, such as somatostatin and gastrin [27], which play crucial roles in regulating gastric motility. Additionally, *H. pylori* ‐induced inflammation may disrupt the enteric nervous system, leading to dysregulation of gastric motor function [28].

Established guidelines for routine *H. pylori* testing and treatment during pregnancy are lacking. However, the American College of Obstetricians and Gynecologists suggests considering it for patients with symptoms resistant to standard antiemetic therapy [29]. Case reports and small series have reported improvement in symptoms among patients with severe disease after treatment of *H. pylori* infection [30]. These findings highlight the need for further investigation into the optimal timing, safety, and efficacy of *H. pylori* testing and eradication therapy during pregnancy.

While certain medications used to treat *H. pylori* , such as bismuth, fluoroquinolones, and tetracycline, are potentially unsafe during pregnancy, other agents used in eradication regimens are considered low risk, particularly after 14 weeks of gestation [30]. Indeed, several case reports and small series have reported symptomatic improvement in patients with severe diseases after *H. pylori* eradication [1, 30]. Prospective studies are needed to investigate whether *H. pylori* eradication therapy leads to an improvement in HG symptoms.

If eradication therapy is deemed necessary during gestation, a regimen comprising amoxicillin, metronidazole, and a proton pump inhibitor is one suggested option that may be considered after the first trimester, as this combination is reported to be relatively safe during pregnancy [30]. It is also important to note that the efficacy of eradication therapy may be lower during pregnancy due to physiological changes and altered drug metabolism. Therefore, it is important to confirm successful eradication after treatment.

A more proactive strategy may be preconception care. Our findings highlight the value of shifting the focus from treatment during pregnancy to prevention before conception. The importance of this approach is further underscored by emerging evidence linking *H. pylori* infection not only with HG but also with challenges in conception itself. A meta‐analysis, for instance, demonstrated a significant association between *H. pylori* infection and female infertility, suggesting it may be a modifiable risk factor, particularly in cases of unexplained infertility [31]. Given these potential dual impacts on both fertility and pregnancy health, these findings suggest the potential value of considering *H. pylori* screening and eradication as a component of preconception counseling, particularly for women residing in high‐prevalence regions such as Japan.

We acknowledge that the idea of preconception *H. pylori* screening and eradication remains speculative. Our observational findings raise the possibility of a proactive strategy, but cannot support recommendations for preconception intervention. The feasibility of such an approach would require a re‐evaluation of national guidelines and insurance policies to support preventative measures. We believe this concept should be framed cautiously as a hypothesis for future prospective trials to validate its efficacy and cost‐effectiveness. Further research is warranted to confirm the benefits and cost‐effectiveness of such an intervention.

This study has several limitations that warrant consideration. Firstly, the reliance on anti‐ *H. pylori* IgG antibody titers as a marker of *H. pylori* infection has inherent limitations. While a positive IgG antibody test indicates past or present infection, it does not differentiate between active infection and previous exposure that has been eradicated, either spontaneously or through treatment. Therefore, some individuals identified as anti‐ *H. pylori* IgG‐positive in our study may not have had an active infection at the time of hospitalization. The reported seroprevalence of *H. pylori* antibodies among individuals who had cleared infection varies, and the precise proportion of those with persistent active infection within this population remains unclear. However, it is also recognized that a substantial percentage of seropositive individuals (possibly ranging from 20% to 50%) may indeed still harbor the bacteria [32] though our IgG testing alone cannot differentiate active from past infection. Future studies employing more accurate diagnostic methods for active infection, such as the urea breath test or stool antigen test, are needed to confirm the association between current *H. pylori* infection and prolonged HG.

In addition to these limitations, the retrospective nature of our study did not allow for the analysis of other potential confounding factors, such as socioeconomic status, psychological comorbidities, or subtle temporal changes in patient management and care protocols over the 13‐year period.

While this study used a weight loss of 5% or more from pre‐pregnancy weight as a criterion for hospitalization, it did not include a detailed analysis of the association between the degree of weight loss and factors such as *H. pylori* antibody status or the length of hospital stay. Weight loss is one of the most important and objective indicators for evaluating the severity of HG, and the lack of this analysis is one of the other limitations of our study. Although daily weight can be influenced by various factors, making it challenging to rigorously assess temporal changes during hospitalization.

This study has highlighted the potential association between *H. pylori* infection, as indicated by anti‐ *H. pylori* IgG antibody positivity, and prolonged hospitalization in women with severe HG. The multivariate analysis identified anti‐ *H. pylori* IgG positivity as a significant risk factor for prolonged hospitalization, suggesting that a patient's serological status, whether due to a current or past infection, is predictive of a more severe clinical course. Our findings suggest that anti‐ *H. pylori* IgG testing, along with assessment of urine ketone levels, may be a useful tool in identifying patients at higher risk for a protracted course of illness. A comprehensive approach that considers not only *H. pylori* infection but also nutritional status, psychological well‐being, and other potential contributing factors is essential for optimizing the management of severe HG and improving maternal and fetal outcomes. Ultimately, a deeper understanding of the complex interplay of these factors will pave the way for more targeted and effective interventions for women suffering from this debilitating condition.

## Author Contributions

**Ryuhei Kurashina:** conceptualization, funding acquisition, investigation, data curation, formal analysis, project administration. **Masafumi Toyoshima:** writing – original draft, methodology, investigation, visualization, validation, project administration. **Jun Ogawa:** data curation. **Youhei Tsunoda:** data curation. **Nozomi Ouchi:** data curation. **Mirei Yonezawa:** data curation. **Rintaro Sawa:** supervision. **Yoshimitsu Kuwabara:** writing – review and editing. **Shunji Suzuki:** supervision.

## Conflicts of Interest

Dr. Yoshimitsu Kuwabara is an Editorial Board member of JOGR Journal and a co‐author of this article. To minimize bias, Dr. Yoshimitsu Kuwabara was excluded from all editorial decision‐making related to the acceptance of this article for publication. The other authors declare no conflicts of interest.

## Supporting information


**Dataset S1:** Raw data used for statistical analysis.

## Data Availability

The data that support the findings of this study are available in Supporting Information of this article.
